# The American and Colonial Journals: Surgery

**Published:** 1839-10

**Authors:** 


### SURGERY.

### *Cases of Sensitive Tumours of the Female Urethra, with Remarks.*

By ALEXANDER E. HOSACK, M.D.

IN May, 1835, I was consulted by a servant woman, in a family where I was in attendance, for a complaint which she said had caused her considerable distress, and, as she expressed herself, it appeared as if something had dropped into the passage immediately after making water, causing her great pain at the moment, and which frequently bled, particularly upon being touched by her linen. Upon the slightest exertion she was seized with bearing-down pains to such a degree as to compel her to take to her bed. These difficulties, she said, had been gradually increasing upon her for two or three years, and being unmarried, she was from delicacy induced to conceal her sufferings until no longer able to bear them.

From this statement I was induced to make an examination, which clearly explained the cause of all her trouble. I discovered two or three little tumours immediately within the meatus urinarius, to which they were attached by a narrow neck. They were of a florid red colour, and appeared to be covered by the delicate lining membrane of the urethra. They were exquisitely sensitive and bled upon the slightest touch. In form they resembled a split pea, varying from that in size to a small kidney bean, and placed upright, in such a manner, as to break the flow of urine. The patient did not however complain of the pain upon urinating as her greatest distress, for it was not to be compared to that caused by exertion, or from contact of the dress, which was frequently excruciating.

By raising these tumours with a probe, I discovered their attachment to be limited to the margin of the urethra, and suggested to her the propriety of having them removed, which I assured her could be readily done, and with comparatively little pain. Having obtained her consent, I snipped them off with scissors: the hemorrhage was not excessive. In a few days the part was healed, and she appeared to be completely rid of the evil, until about six weeks after, when the sensitiveness and other symptoms returned. In the course of three months I was again requested to relieve her if possible by a further operation. Upon examination, I found the margin of the urethra fringed with the same highly-organized structure. It appeared as if the lining membrane had been prolapsed and was turgid with blood; or in other words, had shot out like a fungus. Under these circumstances I determined to remove the diseased structure by excising the meatus urinarius, and this was accordingly done. The wound in due time was healed, leaving the parts apparently sound, with the exception of a few spots of discoloration in the folds of the nymphæ, which I afterwards destroyed by caustic. The extremity of the urethra remaining somewhat harder than might have been expected in sound parts, I expressed doubt whether it might not be the incipient stage of scirrhus. The disease, however, in the course of a few months returned with all the distressing symptoms as before enumerated. The patient being again willing to submit to any operation that I might advise, I determined to remove the urethra to an extent that would hold out a better prospect of success. My friend, Dr. Wilkes, with whom I consulted, confirmed this opinion, and assisted me in the operation. The patient being placed upon the bed in a recumbent position, with the legs flexed upon the body, I began with measuring the length of the urethra, by introducing the female catheter, and marking it the instant the urine began to flow; this precaution I considered necessary, from the fact that the length of the urethra, in females, is very variable; at the same time, I was unwilling to encroach too much upon the bladder, which might endanger consequences more distressing to the individual than the existing disease. The preliminaries being attended to, I seized the fungous excrescence with the *pince* of *Museux*, and drawing it out, I circumscribed the urethra with a knife, carried on the dissection until I had detached about three quarters of an inch in extent, as I supposed. I then examined the urethra at the upper extremity of the wound, and finding it perfectly natural and free from all hardness, I separated it at that point. The hemorrhage for the moment was very great; but by pressure, constantly kept up with a compressed sponge, it was arrested, or so much restrained, as to do away with all anxiety on that account. The patient having made water a short time previous to the operation, I did not consider it necessary to leave a catheter in the bladder, which I afterwards regretted, as I was obliged to draw off the urine the following morning, but not without considerable difficulty, as may be imagined. I determined, however, for the future, to leave the catheter in the bladder, or at least until the urine should flow at its side; which took place on the sixth day, when I removed the instrument. Since which time she has enjoyed full control over that organ, and voids urine with comparative ease.

It is now six months and no return of the disease. No bougie was introduced to keep open the mouth of the urethra, as might *a priori* have been considered necessary. Indeed, I purposely avoided using it, lest the irritation might predispose the parts to a return of the disease. Upon examining the part removed, I found the urethra to be very much thickened and hardened at its extremity, but this circumstance not being observed in other instances of this disease as related by different authors, I must conclude that it had no agency in the growth of these tumours, but was probably the result of irritation.

I first met with this disease in the practice of my friend, Dr. Mott, who, several years ago, was consulted by a gentleman on account of his daughter, who laboured under this distressing complaint. The case was one of great interest, both from the circumstance of the patient being at the delicate age of eighteen, and on the eve of marriage. She had suffered from this disease for two years and upwards, and, considering it an insurmountable objection to marrying, had frequently deferred the nuptial ceremonies, at the same time not willing to break off her engagement; and, unable any longer to conceal her actual situation, she disclosed the true cause to her father, the only surviving parent, who immediately came to New-York, and placed her under the care of Dr. Mott.

In this case, Dr. Mott, after carefully examining the disease, determined upon removing the *meatus urinarius*, to the margin of which two or three small flattened and vascular tumours were attached. They were of the size of small beans, highly florid, and exquisitely sensitive. The wound healed kindly after the operation; the result was perfectly successful, when she returned home to her friends and afterwards married.

REMARKS. Although this disease is one of comparatively rare occurrence, much has been written upon it; still, elaborate works on surgery and midwifery have not, with but one or two exceptions, in any way noticed its existence. I confess it appeared to me to be quite a novelty; and as regards the excising of part of the female urethra for its removal, or for any other object, I do not recollect ever to have heard of a single instance; nor have I yet been able to discover that it has been done to any extent, beyond the mere margin of the external orifice of the urethra. This disease is first spoken of by Morgagni, who, under the head of excrescences and other diseases of the female urethra, remarks: "examining the body of an old woman, about the beginning of 1751, I met with a small triangular excrescence within the external orifice of the urethra, but it was not prominent;" and, in another part of the same chapter, he goes on to state, "that there is a red and fungous excrescence, which is of the size of a bean, sometimes to be observed attached to the orifice of the urethra." This disease is also described by an Englishman by the name of Hughes, of Stroudwater, in Gloucestershire, in 1769. In a case described by that gentleman, he speaks of it as of "a red colour, and of a softish, spongy texture, with an irregular, jagged surface; was sore when touched; and a bloody serum oozed from it." The patient was eleven years of age, and of a very thin habit of body; it had existed for three years. In this case, Mr. H. removed the *meatus urinarius*, which completely included the disease. The patient suffered for some time from retention of urine; only, however, during the healing process. Five years had elapsed since the operation, and the patient continued perfectly well.

"On examining the fungus, after the operation, it appeared about the size of the nipple of an adult; its anterior part being expanded, formed, as it were, a little cup, with its border indented like a cock's comb, having a hole in its bottom which was the orifice of the urethra, which ran through the body of the fungus; the internal membrane of the urethra was continued to the edge of the indented border, which was of a deeper red colour and softer texture than the other part of it." In volume xiii. page 784 of the Lancet, Mr. Wardrop has published four cases of this disease. The first was in a young girl thirteen years of age; the second in a lady of thirty years of age; the age of the third is not given; the fourth was upwards of sixty years old. In all of these cases Mr. Wardrop speaks of the exquisite sensitiveness as the most prominent symptom. In one of the cases above alluded to, the disease returned after marriage, when the patient again applied to Mr. W. for relief. He states "that the tumour had now assumed the appearance of a bright scarlet fungus, encircling the meatus, and was attended with such exquisite tenderness as to prevent sexual intercourse. The orifice, including the disease was removed, and it did not return."—Boyer makes mention of a fungus occurring in the female urethra. It is however noticed in a more particular manner by Bromfield, and according to Mr. Hughes, accounts are also given of it by Sharp, Warner, and Jenner. Mr. Wardrop also refers to Chaussier and Dubois, and states that it is particularly noticed by Madame Lachapelle as well as by Rosenmuller, Vogel, Kaldibrand, Drochaska, and some other German pathologists. In many of the instances referred to by the older writers, the symptoms were at first mistaken for those of stone in the bladder, and in the case just related by Mr. Hughes, the disease was mistaken by those who were first consulted for prolapsus of the uterus, and actually treated as such, nor was the error discovered until the patient was unable any longer to bear the pain consequent upon the pressure applied to that viscus.

By reference to the foregoing cases we arrive at the following conclusions:

1st. That the disease is characterized by peculiar symptoms.

2d. That it is not confined to any age.

3d. That it is unaccompanied with discharge, unless the parts be chafed or abraded.

4th. That, in order to prevent a return of the disease, it is better to remove, at once, the external orifice of the urethra, including the tumours.

5th. That it is a complaint of slow growth, and does not attain to any great size; for, in no instance yet recorded, so far as I am enabled to learn, has it been found larger than a small cherry.

*Note.* The only inconvenience to be expected from removing the meatus urinarius, will be that of retention of urine. This is not, however, a necessary consequence; for among all the authorities quoted, but two cases are mentioned in which it occurred; one related by Mr. Hughes, and the other by myself. In both instances they were spontaneously relieved, on the fifth or sixth day.

[The only remark we would make on the above interesting communication, is to express a doubt whether the less formidable operation of the destruction of the parts by some form of caustic should not have had a trial.]

*The New York Journal of Medicine. July,* 1839.

*** This is the first number of a new Quarterly Journal, published in New York, by our own highly respectable publisher, Mr. George Adlard. The present specimen augurs well for the future importance of the work, to which we wish every success. The plan of the journal is good; its contents highly creditable to the contributors; and the *getting-up* equal to that of our best English periodicals. It is about one fourth or one fifth less in size than our own Journal, and sells for about one sixth less price. We hope often to profit by its original articles.

---

### *Views and Treatment of an important Injury of the Wrist.*
By J. Rhea Barton, M.D.

The accidents which are to be the principal subject of my remarks, usually pass either for sprains or dislocations of the wrist. Under one of these denominations are these cases to be detected, which, though partaking somewhat of the character of sprains or dislocations, are distinguishable from either of them respectively. They may be recognized by their being accompanied by more distortion of the hand and arm than any which can arise from simple sprains of the wrist, and yet less than that which must necessarily take place when there exists a complete luxation of the carpus. The profile of the limb under this injury is a peculiar one, distinguishing it on the one hand from the sprained wrist, and on the other from luxation.

In simple sprains of the wrist, though accompanied by extreme swelling, the limb will still be found to retain a characteristic outline of its natural contour. It is not marked by any abrupt and solid eminences, the swelling is rather uniform, diffuse, and puffy, the hand continues on the same line with that of the forearm, &c. In complete dislocations, the nature of the injury must always be very palpable from the great bulging of the overlapped bones, and from the shortening of the limb, &c. Between these two injuries, there is too great a dissimilarity to admit of an excuse for the surgeon who mistakes the one for the other: but he may confound with these, and it is a common fault to do so, *a sub-luxation of the wrist, consequent to a fracture through the articular surface of the carpal extremity of the radius;* although to this accident belong appearances exclusively its own. It is to this peculiar injury that I wish to draw attention.

It is one of the most common injuries to which the upper extremities are subjected; and every practitioner of moderate experience will, I am sure, be able to call to his recollection the appearance which the limb presents under such circumstances, as well as the embarrassment which he has experienced in his attempts to obviate eventual deformity, to preserve the functions of the fingers, and to restore the motions of the wrist and forearm.

The similarity of manner in which this accident generally occurs is striking. It is almost always found to have taken place in consequence of the individual having thrown out his hand to rescue himself from falling, or to ward off injuries threatening a more important part of the body. In the act of falling, for example, the hand is thus instinctively thrown out, and the force of the fall is first met by the palm of the hand, which is violently bent backwards until the bones of the wrist are driven against the dorsal edge of the articulating surface of the radius, which, being unable to resist, it gives way. A fragment is thus broken off from the margin of the articular surface of this bone, and is carried up before the carpal bones, and rested upon the dorsal side of the radius; they having been forced from their position, either by the violence or by the contraction of the muscles alone. We have then an imperfect luxation of the wrist, depending on a fracture through the extremity of the radius. The deformity will be found to correspond with the state of the case. There is a tumour on the dorsal side of the arm formed by the bulging of the carpal bones and fragments; whilst below it, on the palmar side, the extremity of the radius projects. The degree of prominence of these parts, depends upon the size of the fragment and the violence of the injuring force. The ulna not being very intimately involved in the injury, retains its position, and serves as an abutment, against which the hand seems to rest; whilst the radius, as it has its edge broken off, allows the hand on that side to be drawn upward, and hence to render, on the under side, the styloid process of the ulna more conspicuous than natural. Crepitus cannot always be felt, sometimes in consequence of the smallness or crushed condition of the fragment; at other times, owing to the great swelling and tension; but in every such case, the distortions of the limb are to be seen, and may be removed by making firm extension and counter-extension from the hand and elbow, at the same time gently depressing the tumours already spoken of. By the employment of these means, all deformity, except that which evidently depends upon the more general swelling, may be satisfactorily removed; but the moment the extension and counter-extension are relaxed, the combined action of the flexors and extensors of the fingers, as well as those of the wrist, force the deformity to reappear as conspicuously as before: and as often as the effort is renewed and discontinued, will the deformity appear and disappear. In this respect does this species of injury in an especial manner differ from a complete simple luxation of the wrist, which, when once reduced, must continue so after the reducing force has been withdrawn. There is no spontaneous reluxation after the simple complete dislocation has been removed; whereas, in this case it immediately succeeds the withdrawal of the force. This accident must not be confounded with those which are also of frequent occurrence, namely, fracture of the radius, or of the radius and ulna just above, and not involving the joint. It will be found, on referring to the writings of Boyer, Desault, Sir Astley Cooper, Dupuytren, and many others, that this frequently happens, and that the fracture often reaches to within a few lines of the extremity of the bone; and that these cases are very frequently mistaken for dislocations, though they are in reality fractures exterior to and disconnected with the joint; the deceptive deformity being occasioned by the displacement of the broken ends of the bone caused by the action of the muscles and the weight of the hand.

The fragment may be, and usually is, quite small, and is broken from the end of the radius on the dorsal side, and through the cartilaginous face of it, and necessarily into the joint. The pronator quadratus is not involved in the fracture. The radius and ulna are not materially disturbed in their relations to each other The only important change, which takes place in consequence of this fracture is, that the concave surface at the extremity of the radius, which receives and articulates with the first three carpal bones, is converted, as it were, into an oblique surface by the loss of a portion of its marginal ridge; commonly by the separation of an entire piece; sometimes by the crushing of its substance. The moment the cartilaginous extremity of the radius is deprived of its concave form, the united force of the carpal and digital flexors and extensors is exerted to create a complete luxation; but as the ligaments are only stretched, or but partially torn, this cannot take place. The carpal bones, therefore, only emerge collectively from their natural position, and carrying before them the broken piece, rest on the dorsal side of the radius, forming a tumour there; whilst the end of the radius itself occasions on the palmar side a prominence which is round and smooth, and differing in this from similar projections formed by the fractured ends of bones, the abruptness and harshness of which may sometimes be distinctly felt through the soft parts, and which are themselves, when pressed upon, acutely painful.

It follows of course in injuries of this kind, that unless some method of dressing be adopted whereby the retraction of the hand may be permanently counteracted, and the prominences repressed, the patient will recover with a crooked arm, and under a sacrifice of some of the functions of the hand. The customary modes of treating either sprains or dislocations of the wrist, or fracture of the forearm, are totally inadequate to the purpose, and should not be relied on as a treatment for these particular cases by any practitioner who has regard for the welfare of his patient, and for his own reputation. There is no professional point upon which I can more confidently express myself, than upon the errors committed in the treatment of these cases,—passing, as they commonly do, for sprains of the wrist, and hence treated as such. After an unvarying success in the management of this accident for many years in the Pennsylvania Hospital, in the Blockley Hospital, and in private practice, I can strongly recommend the following plan of treatment: Two thin, but firm splints of wood, are to be prepared, of sufficient length to extend from just below the condyles of the os humeri to the ends of the fingers, and of width enough to embrace the sides of the limb. These are to be lined on one of the sides with carded cotton, or something equally soft, and wrapped with a bandage. Two compresses, each about two inches square, and composed of strips of bandage, about one yard and a half long, evenly folded up, are also to be in readiness. The arm is then to be flexed at the elbow, and one assistant is to hold it firmly above the condyles, whilst another makes extension from the fingers. The surgeon now presses the prominent end of the radius on the inner side, and the bulging carpus and fragment on the outer side, into their respective places. The roller is then to be lightly pressed around the hand and arm, securing in its course up the limb one of the compresses precisely over the carpus and back of the hand, and the other with equal precision over the palmar side of the radius just above its carpal extremity. These compresses, when properly arranged, will be found *not opposite to each other, but the inner one commencing on a line opposite to that on which the outer one has terminated.* These being applied, the inner splint is next placed against the limb,—the assistant shifting his hand to admit of this being done, without his relaxing in the least degree the extension until the limb is bandaged to this splint, when it will be found that the extension is well maintained. The outer splint is now to be applied and secured to the arm by the return of the roller. The principal use of the latter splint is to act upon the outer compress, and by its general pressure to weaken for the time the force of the resisting muscles. By the employment of these simple means, the indications in the treatment of this accident will be found to be fully met. The arm may be carried in a sling, and the patient permitted to walk about, &c. In three or four days the limb should be undressed and inspected; and whilst held so that relaxation cannot take place, the wrist and fingers are to be bent enough to preserve the flexibility of the joints. The dressings are then to be reapplied. These operations are thenceforward, for four or five weeks, to be repeated every day, adding to them the motions of pronation and supination.

The practice of keeping a limb in splints, with the joints in an immoveable state for weeks, even when the fracture is remote from the articulation, cannot be too earnestly deprecated; and in cases where the injury to be repaired has involved a joint, such treatment is censurable to a high degree, as it is almost certain to destroy the mobility of it by promoting the adhesion of ligaments, the union of tendons with their thecæ, and by obliterating bursæ—evils never to be fully repaired. *Philadelphia Med. Examiner. Nov.* 7, 1838.

---

*On the Use of Iodine in Herpes.* By G. BILLINGSLEA, M.D.

FOR the last twelve years I have used the alcoholic tincture of iodine as a local application in those troublesome cases of herpes circinatus or ringworm so common in our southern country, with the happiest effect. Indeed I do not recollect any case in which it has been used without a radical cure. As I believe it is not generally known to our medical public, not having seen it recommended by any author in similar cases, you may confer a favour on some of the readers of your valuable periodical by inserting this notice.

*American Med. Intelligencer. May*, 1839.

